# Eating soup with nails of pig: thematic synthesis of the qualitative literature on cultural practices and beliefs influencing perinatal nutrition in low and middle income countries

**DOI:** 10.1186/s12884-016-0991-z

**Published:** 2016-07-28

**Authors:** Shanti Raman, Rachel Nicholls, Jan Ritchie, Husna Razee, Samaneh Shafiee

**Affiliations:** 1 School of Public Health & Community Medicine, University of New South Wales, & South Western Sydney Local Health District, Health Services Building Level 3, Cnr Campbell & Goulburn St, Liverpool, NSW 2170 Australia; 2 Faculty of Health, University of Technology, Sydney Level 7, 235 Jones St, Ultimo, NSW 2007 Australia; 3 School of Public Health & Community Medicine, University of New South Wales, Samuels Building, Gate 11, Botany Street, Randwick, UNSW, Sydney, NSW 2052 Australia

**Keywords:** Maternal health, Low and middle-income countries, Maternal and child nutrition, Breastfeeding, Culture, Traditional practices, Qualitative research, Synthesis

## Abstract

**Background:**

The perinatal period, i.e. pregnancy, childbirth and early infancy, is a significant transition period where the biological and the social strongly intersect. In low and middle-income countries the disease burden arising from the perinatal period, is still substantial. The perinatal period is also a crucial window of opportunity for reducing undernutrition and its long term adverse effects.

**Methods:**

We explored qualitative research conducted in low resource settings around the perinatal continuum over the past two decades, with a particular focus on the ‘cultural’ realm, to identify common themes influencing maternal and infant nutrition. We systematically searched electronic databases from 1990 to 2014, including MEDLINE, EMBASE, PsycINFO, Scopus and Cumulative Index to Nursing and Allied Health Literature, using relevant search terms including traditional beliefs, practices, pregnancy, childbirth, developing countries etc. Adapted Consolidated Criteria for Reporting Qualitative Health Research and Critical Appraisal Skills Programme criteria were used to determine quality of studies. We synthesised the literature thematically, enabled by NVivo 10 software.

**Results:**

Most studies showed cultural support for breastfeeding, although most traditional societies delayed breastfeeding due to colostrum being considered ‘dirty’. A range of restrictive practices through pregnancy and the post- partum period were revealed in Asia, Latin America and Africa. There was a strong cultural understanding of the healing power of everyday foods. A wide range of good foods and bad foods continued to have currency through the perinatal continuum, with little consensus between groups of what was beneficial versus harmful. Cross-cutting themes that emerged were 1) the role of the woman/mother/wife as strong and good; 2) poverty restricting women’s nutrition choices; 3) change being constant, but the direction of change unpredictable.

**Conclusions:**

A rich and diverse repertoire of cultural practices and beliefs influenced perinatal nutrition. Results from this synthesis should influence public health policymakers and practitioners, to tailor contextually specific, culturally responsive perinatal nutrition interventions to optimise health and wellbeing of mother-infant dyads. Ideally these interventions should build on culturally sanctioned life affirming behaviours such as breastfeeding, promoting post-partum rest and recovery, while modifying the potentially harmful aspects of other cultural practices in the perinatal period.

**Electronic supplementary material:**

The online version of this article (doi:10.1186/s12884-016-0991-z) contains supplementary material, which is available to authorized users.

## Background

Health conditions affecting the perinatal period account for a major contribution to disease burden in sub-Saharan Africa and South Asia [1, 2], making the perinatal period a key period for health intervention. Despite this understanding there remain major gaps and disparities, with the burden of maternal and child mortality, morbidity and malnutrition occurring largely in resource poor settings. Many international studies have highlighted the importance of maternal factors such as education levels, health, and nutrition in infant and child outcomes [3–5], upstream social determinants such as poverty, illiteracy, poor status and care of women are also known critical underlying factors [6, 7]. A major review of what “works” in maternal and child health in the developing world found a paucity of good quality community-based data from developing-country studies [8].

In the post millennium development goal (MDG) era, maternal and child undernutrition continue to be problematic contributing to 800 000 neonatal deaths, stunting, wasting, micronutrient deficiencies, and nearly three million child deaths annually [9]. There are significant barriers at the household level in accessing appropriate nutrition for women and children [10]. The period from in-utero to 24 months of age, i.e. the perinatal period, is a crucial window of opportunity for reducing undernutrition and its adverse effects [11]. While pregnancy and childbirth are natural health transitions; biophysical, psychosocial, cultural, and social factors are integral to the perinatal experience and impact on outcomes. There is robust evidence from diverse settings that health care seeking [12, 13], maternal [14], and child survival [15] can be influenced by socio-cultural factors.

There is an emerging body of research that illuminates the ways in which maternal dietary and infant feeding practices relate substantially to local cultural norms and constraints [10, 16, 17]. We therefore aimed to explore qualitative research conducted in low resource settings around the perinatal continuum, with a particular focus on the ‘cultural’ realm of shared beliefs, values, and practice; and to identify common themes in the research-base. Our understanding of culture was deliberately broad and drew upon both “transmitted pattern of meanings embodied in symbols, a system of inherited conceptions expressed in symbolic form” [18], and “complex integrated system of thought and behaviour shared by members of a group” [19]. We focused on the period 1990 to 2014, incorporating the years of accountability of the MDGs, to explore change in cultural practices over the decades.

Given the established burden of maternal and child morbidity and mortality in low resource settings, this review focused on published qualitative studies from low and middle-income countries (LMICs) that explored cultural beliefs and practices influencing perinatal nutrition for mother and infant. The review is part of a larger synthesis exploring wider cultural practices and beliefs influencing perinatal health and wellbeing.

## Methods

We chose to limit our review and synthesis to qualitative research as we were focusing on cultural beliefs, practices; and wanted to mine rich primary data. Interpretive synthesis of qualitative research is a distinct methodology with distinct benefits, offering the potential for insight, vividness and significantly inform policy and practice [20, 21]. We sought studies presenting primary data and involving purely qualitative or mixed method data collection techniques (interviews, focus groups, ethnography) reporting on cultural or traditional practices and beliefs influencing the perinatal period (i.e. pregnancy, childbirth and infancy) in LMICs. We excluded studies from countries not classified by the World Bank as LMIC [22]. We also excluded articles which had no information on cultural practices/beliefs, had no qualitative data, or no primary data; those specifically focussed on high risk conditions such as Human immune-deficiency virus (HIV), diabetes, post-partum haemorrhage, perinatal loss and anaemia. Non-English articles were excluded to prevent cultural and linguistic bias in translations.

We systematically searched the following electronic databases from 1990 to 2014: MEDLINE, EMBASE, Cochrane Library, PsycINFO, Cumulative Index to Nursing and Allied Health Literature (CINAHL), Science Citation Index, WHO Global Health Library, Scopus, Web of Science and reference lists of relevant articles. An example of the search strategy used for electronic databases including MEDLINE and EMBASE was: (perinatal OR pregnancy OR childbirth OR antenatal OR postnatal OR infancy) AND (“traditional beliefs” OR “cultural beliefs” OR “cultural practices”) AND (“developing countries” OR “low income” OR “low resource setting”) AND (qualitative OR “focus group” OR interviews OR ethnography). We also searched Qualitative Health Research, Qualitative Research, Social Science and Medicine, Qualitative Inquiry and Google Scholar. Hand searching complemented electronic searches. Searches were conducted using the following individual and combined keywords: perinatal, pregnancy, childbirth, antenatal, postnatal, infancy, traditional beliefs, cultural beliefs, cultural practices, developing countries, low income, low resource setting. The search was conducted between 6 June to 12 August 2014, and followed the preferred reporting items for systematic reviews and meta-analyses (PRISMA) guidelines where applicable [23], see attached PRISMA checklist (Additional file 1).

Two reviewers (SR and RN) screened the abstracts, discarding studies that did not fit the inclusion criteria. The full-text versions of retrieved studies were analysed and examined for study eligibility with uncertainties and disagreements being resolved in consultation among the reviewers. Two authors (SR and RN) independently assessed the explicitness and comprehensiveness of reporting (i.e. quality of studies) by using an adapted consolidated criteria for reporting qualitative research framework (COREQ) [24], scored out of 12, and the CASP Qualitative Research Checklist [25], scored out of 10.

We performed a synthesis of the studies that met the inclusion criteria, based on the J Thomas and A Harden [21] ‘thematic synthesis’ method, which draws on the principles of primary qualitative research. For each paper with primary data, i.e., from interviews, focus groups, ethnography, we extracted all the participant quotations and descriptive text under the “results/findings” or “conclusion/discussion” section of the article. These were entered verbatim into NVivo 10 [26]. Our process of thematic synthesis involved line-by-line coding of the findings of the primary studies, organizing the codes into descriptive themes, and abstracting the findings to produce a new interpretation, which went beyond the original studies [21].

## Results

**Fig. 1:**
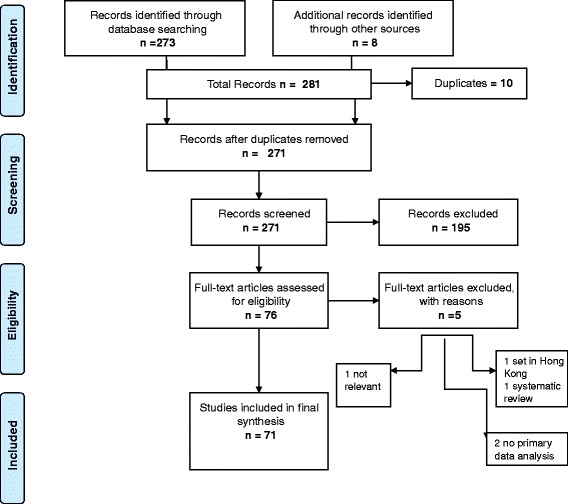
Flow diagram of search and study inclusion process

Our initial search revealed 273 papers. After 10 duplicates were removed, 263 remained, but 195 were excluded primarily because they did not report qualitative research. An additional eight articles were identified from hand searching. Of the 76 eligible studies, five were further excluded, leaving a final list of 71 (see Fig. 1). Table 1 lists studies by region, author, year published and quality scores. Most studies were from Africa and Asia and the majority were published after 2000 (Table 2).

**Table 1:** Included studies by region, author, year and quality scores

Setting	Author (Year)	CASP^a^ Score	COREQ^b^ score
Africa			
Nigeria, rural	Asowa-Omorodion (1997) [74]	7	8
Uganda, rural	Ayiasi et al. (2013) [34]	8	10
Mozambique, peri urban	Chapman (2003) [75]	9	9
Ghana, urban	Dako-Gyeke et al. (2013) [42]	9	8
Ethiopia, rural	Degefie et al., (2014 ) [76]	8	6
Burkina Faso, rural	Donmozoun et al. (2014) [77]	6	4
Sierra Leone, rural & urban	Dorwie and Pacquiao (2014) [78]	10	10
Nigeria, rural & urban	Ejidokun (2000) [55]	10	10
Ghana, rural & urban	Farnes et al. (2011) [38]	10	10
Ethiopia, rural	Gebrehiwot et al. (2012) [79]	9	7
Kenya, rural & urban	Geissler et al. (1999) [45]	7	6
Tanzania, rural	Gross et al. (2013) [80]	8	10
Uganda, rural	Kwagala (2013) [57]	9	6
Uganda, rural	Kyomuhendo (2003) [81]	7	5
Liberia, rural	Lori and Boyle (2011) [82]	10	11
Zambia, rural & urban	Maimbolwa et al. (2003) [41]	10	10
Tanzania, urban	Mbekenga et al. (2013) [72]	9	9
Ghana, rural	Mills and Bertrand (2005) [36]	9	10
Ghana, rural	Moyer et al. (2014) [13]	9	10
S Africa, rural	Ngomane and Mulaudzi (2012) [83]	9	9
Nigeria, rural	Orisaremi (2013) [35]	9	7
S Africa, rural	Preez (2012) [84]	8	10
Ghana, peri urban	Theroux et al. (2013) [58]	7	11
Swaziland, rural	Thwala et al. (2011) [62]	7	9
Swaziland, rural	Thwala et al. (2012) [85]	8	10
Cameroon, rural	Thwala et al. (2012) [86]	9	9
Ethiopia, rural & urban	Warren (2010) [87]	6	6
Ghana, rural	Wilkinson and Callister (2010) [61]	9	8
Malawi, rural	Zulu (2001) [88]	8	8
South Asia			
Bangladesh, urban	Ahmed et al. (2010) [60]	9	7
India, rural	Bandyopadhyay (2009) [31]	7	9
Nepal, peri urban	Brunson (2010) [73]	9	11
Bangladesh, rural	Choudhury and Ahmed (2011) [89]	9	9
Bangladesh, urban	Choudhury et al. (2012) [54]	8	8
Pakistan, rural	Dykes et al. (2012) [29]	7	7
Pakistan, urban	Fikree et al. (2004) [51]	8	4
Pakistan, urban	Fikree et al. (2005) [16]	7	6
India, rural	Iyengar et al. (2008) [90]	8	8
Nepal, rural	Kaphle et al. (2013) [63]	10	10
India, rural	Kesterton and Cleland (2009) [91]	8	10
Pakistan, rural	Khadduri et al. (2008) [33]	7	6
Bangladesh, urban	Moran et al. (2009) [92]	9	8
Pakistan, rural	Premji et al. (2014) [37]	10	11
Bangladesh, urban	Rashid (2007) [93]	8	8
India, rural	Sharma et al. (2013) [94]	10	10
Nepal, rural	Thapa et al. (2000) [52]	7	6
Bangladesh, rural	Winch et al. (2005) [32]	8	7
Asia, other			
Tibet, rural	Adams et al. (2005) [43]	10	9
Laos, rural	Alvesson et al. (2013) [95]	9	8
Laos, rural	de Sa et al. (2013) [27]	8	8
Turkey, urban	Ergenekon-Ozelci et al. (2006) [30]	7	5
Vietnam, rural	Graner et al. (2013) [96]	10	10
Philippines, peri urban	Hadwiger and Hadwiger (2012) [50]	9	10
China, urban	Kartchner and Callister (2003) [97]	9	8
Laos, urban	Lee et al. (2013) [28]	10	9
Vietnam, urban	Lundberg and Ngoc Thu (2012) [46]	9	11
Vietnam, urban	Lundberg and Trieu Thi Ngoc (2011) [49]	10	11
Cambodia, rural	Matsuoka et al. (2010) [98]	8	5
China, rural	Raven et al. (2007) [48]	9	11
Myanmar, rural & urban	Sein (2013) [39]	10	8
China, rural	Strand et al. (2009) [53]	9	7
Laos, rural	Sychareun et al. (2012) [99]	8	10
Cambodia, rural	White (2002) [40]	9	7
Cambodia, rural	White (2004) [47]	9	8
Bali, rural	Wulandari and Klinken Whelan (2011) [44]	9	10
Middle East			
Syria, urban	Abushaikha and Massah (2013) [100]	8	11
Jordan, urban	Khalaf and Callister (1997) [101]	8	7
Latin America			
Guatemala, rural	Berry (2006) [102]	8	8
Argentina, urban	Hess and Maughan (2012) [59]	8	6
Brazil, rural	Piperata (2008) [56]	10	9
Guatemala, rural	Radoff et al. (2013) [103]	10	8

^a^ *CASP* Critical Appraisal Skills Programme, scored out of 10

^b^ *COREQ* consolidated criteria for reporting qualitative research, scored out of 12

**Table 2:** Summary of included studies by region, time period and quality scores

Region	(N)	Time frame	(N)	CASP^a^ Scores	COREQ^b^ scores
Africa	29	1990–1999	2	7	6–8
		2000–2009	6	7–10	8–10
		>2010	21	6–10	4–11
South Asia	18	2000–2009	10	7–9	6–9
		>2010	8	8–10	7–11
Asia (other)	18	2000–2009	7	7–10	5–11
		>2010	11	8–10	5–11
Latin America	4	2000–2009	2	8–10	8–9
		>2010	2	8–10	6–8
Middle East	2	1990–1999	1	8	7
		>2010	1	8	11

^a^ *CASP* Critical Appraisal Skills Programme, scored out of 10

^b^ *COREQ* consolidated criteria for reporting qualitative research, scored out of 12

The following themes relating to cultural practices and beliefs influencing perinatal nutrition were identified.

### Breastfeeding: “Everyone here breastfeeds their babies” [27]

Breastfeeding was overwhelmingly supported culturally, but there was wide variation in practice. Several cultural practices supported breastfeeding, including “preparing the breast and cleaning both mother and infant” [27], and massaging fire ash around the breast when wet [28], as in rural Laos. On the other hand, taboos and prohibitions pertaining to breastfeeding were also common. While religious support for breastfeeding was apparently valued, “the ideal breastfeeding duration is up to two years according to Islam” (Pakistani woman) [29], a young Turkish mother had different understanding [30]:

Pregnant women’s milk is spoiled milk. It may give harm to the breastfed baby.. . . I know it because the *Imam* [Muslim priest] said pregnant women should not breastfeed their babies.

Prelacteal feeds were particularly common in South Asia and were believed to have healing and religious properties. Whether in rural India “hot water, sugar-water, honey, mustard oil, tea, or goat/cow milk” [31], or “sugar water (*misri pani*) and banana” in Bangladesh [32] or ‘*Ghutti’* (honey, butter mixed with sugar, glucose and other liquids) across northern India and Pakistan [29]. In Pakistan for example mothers will not breastfeed till an elderly pious person gives the baby *ghutti*; believed to transfer their qualities to the baby [33]. In Uganda, younger mothers tended to “give glucose”, whereas older mothers tended to give “water, sugar, salt or tea” [34].

Beliefs about maternal behaviour influencing the quality of breast milk are illustrated in this quote from elderly women in rural Nigeria, “in the past, a breastfeeding mother could not sleep with her husband because it would cause diarrhoea in her baby.. .but these days there are drugs everywhere” [35]. Prolonged breastfeeding while generally supported in traditional societies, can co-exist with abrupt weaning, as in the Tarok in Nigeria, who believe that pregnancy affects breastmilk making it unhealthy for the child [35]:

In a traditional setting, the child should be up to one year, eighteen months, or two years plus before you stop breastfeeding. When you stop before then it attracts a lot of stories in the community.. . that you want to spoil your child, you want to start sleeping with your husband, and so on.” (Older Tarok woman)

Traditional practices to see if breastmilk is good enough may still be practised in parts of Africa; for example “an ant was placed in the milk and if the ant died, the milk was considered unwholesome” (Ghana) [36].

We found that colostrum was routinely rejected by most traditional societies, although change has been slowly occurring. Colostrum in Turkish dialect was referred to as *mawu* (new milk) or *fro* (dense milk); Turkish women believe that it should not be given to newborns as it caused “stomach ache” [30]. Indeed some women even felt “that colostrum can kill the baby because it is dense, dirty, old milk stored in the breast for 9 months” [33].

I put my son to breast after two days, as the milk ‘comes’ only after 48 hours. Before I put him to breast I squeezed the yellowish liquid and threw it, as it is harmful for his health. (mother from rural India) [31]

Breastfeeding was strongly sanctioned and supported by elders, “my mother was the first one who motivated me to breastfeed, she talked about breastfeeding before the doctor did” (young mother, Vientiane) [28]. However there were differences of opinion between elders and health professionals; elders advised discarding colostrum, delaying initiation of breastfeeding or introduction of other food well before six months [28, 37], whereas health professionals advocated exclusive breastfeeding for six months. Mothers revealed ambivalence about duration of breastfeeding; while breast milk was “best in the first period of birth”, Laotian and other Asian mothers felt that that as the baby grows, breast milk contained insufficient nutrients [28].

### Healing foods and medicines: “God’s own way of helping the baby” [38]

There was widespread belief in the healing properties of certain foods and its use in the perinatal period; especially in more traditional populations in Asia and Africa (see Table 3). In Myanmar, “almost all women did smearing or drinking of turmeric to prevent muscle pain” [39]. Traditional medicines and herbal preparations for the puerperium and to strengthen breast milk were highly prevalent, to “help with expelling lochia and control post-partum bleeding” and to “produce breast milk abundantly.” In Myanmar as in other parts of South East Asia, “licensed indigenous medicine” were available even in hospitals [39]. “If you have blood left inside, you feel weak, … then you find Khmer medicine to drink to make all the blood come out..”; Cambodian women’s perspectives [40]. In parts of Africa where home births were practised, traditional birth assistants’ advice to women included the use of traditional medicines to widen the birth canal and precipitate labour. As this Zambian *mbusa* (traditional birth attendant) explained, “if the labour is prolonged and the woman has confessed that she has been unfaithful to her husband, she should be given traditional medicine so that labour can progress well!” [41].

**Table 3:** Examples of ethno medicine / healing foods used by women during the perinatal period

Author	Region	Food substances	Effect
Adams [43]	Tibet	Butter ingested by newborn	In order for child to have a clear mind and well-developed senses
		*Chang* warm barley beer ingested by mothers	
Ayiasi [34]	Uganda	*Waragi* local alcohol	Generally therapeutic, keeps infant’s skin clear
Farnes [38]	Ghana	Local herbs ingested by mothers	Prevents *sunsumyare* (spiritual sickness), promotes maternal, fetal health, prevents complications
Hadwiger [50]	Philippines	Ginger, carried	Protect unborn baby from *aswang* (evil spirits)
Lundberg [49]	Vietnam	Pig’s trotter with papaya or red bean and potato, meat and eggs	Enrich blood, help recovery, encourage expulsion of the lochia, stimulate lactation
Maimbola [41]	Zambia	Traditional medicine applied to vagina	Prepare and widen the birth canal in pregnant women
Ngomane [83]	S Africa	*Mbita*, *Ritlangi*, *Mpundulo*	Strengthen and preserve pregnancy Induction, management of labour and management of pain
		*Mbheswan*a, roots of *Xirhakarhan*i, boiled *Dinda*	
Radoff [103]	Guatemala	Teas and baths from grasses and trees, cypress, pine, oak, pear, eucalyptus	Stimulate labour, reduce postpartum bleeding
Raven [48]	China	Ginger and wine	Enrich blood, help recovery, encourage expulsion of the lochia, stimulate lactation
		Meat and eggs	
Sein [39]	Myanmar	Turmeric, ingested or applied on skin	Prevent muscle pain and to prevent newborn from abdominal pain
Thapa [52]	Nepal	Mustard oil, turmeric, eggs ingested	Regain energy post-partum, make womb strong, relieve pain
Theroux [58]	Ghana	Bitter leaf, dandelion, *prekos*, *maringa*, *nim* tree, and *kontosi*	Treat minor illness and maintain/improve pregnancy
		*Fou-fou* pounding	Prepare for labour
Wulandari [44]	Bali	Tamarind, turmeric, cinnamon, clove, coconut	Improve maternal and infant health
		Herbal medicines	

In many traditional societies, ethno-medicine was an accepted part of the perinatal experience and herbs were prescribed by a range of religious and healthcare professionals, including herbalists, *akomfo* (witch doctor), Christian pastors and birth attendants. In many parts of Africa herbs promoted women’s sense of autonomy and “can be considered God’s medicine” [38], Ghanaian women reported that “herbal medicine gives strength”, whereas hospital drugs “weakens me when I take them” [42]. Tibetan medical texts prescribed traditional food and drink as rituals for newborn care [43]. In Bali, traditional treatments were also common, according to this informant [44]:

Health-care workers are always thinking that it is medicine that will keep us healthy. The truth is there are many alternatives we can use to make us healthy. One of them is traditional herbs.

A practice reported widely in African studies was soil eating. Attitudes to soil-eating were ambivalent and gendered; women’s soil-eating being more acceptable than men's and only really approved of for pregnant women [45]. Kenyan women believed that soil influences the volume and condition of the blood, which in turn affects the *tsango* (guardian of the body's health and fertility).

### Good food versus bad food: “Eat soup with nails of pig or pork ragout and green papaya every week” [46]

The synthesis revealed a wide range of foods that were considered helpful or ‘good’ versus those that were actively harmful. Across Asia, notions of foods that were considered “heaty” as against those that were considered cooling had widespread currency. In many parts of Asia, post- partum cultural practices necessarily involve restoring heat to the woman’s body; recognising that having lost energy and blood during delivery, both *yin* and *yang* are weakened. Khmer women ate special hot foods such as *Khaw* (a traditional dish of beef), pork, or fish braised with salt, pepper, and palm sugar by women who could afford it [47]. In China, meat and eggs were regarded as “hot”; food could be made “warmer” by adding ginger and wine and “warm” food helped restore balance [48]. Vietnamese cultural practices relating to food were similar; fresh vegetables and fruits were considered ‘cold’, but new mothers were allowed to consume some boiled vegetables [49]. Traditionally Asian women were encouraged to eat high protein or ‘hot’ foods to build up the lost vital energy or *chi’I* [49].

In Vietnam, the most common foods encouraged to stimulate lactation were pig nails with green papaya or red bean and potato, considered helpful in increasing milk production [46]. Almost diametrically opposed was the view from Bali that “it is better if we eat lots of vegetables” because vegetables increase the production and ‘freshen’ the taste of breast milk [44]. In Bengal, post-partum mothers were encouraged to take milk, ghee, butter, and certain types of fish to increase the quantity of breast milk and encouraged to eat garlic believed to enhance the process of “drying of the womb” [31]. In the Philippines, boiled vegetables or *laswa* was promoted in pregnancy to make the mother and the foetus strong. As this quote from a young mother typifies [50]:

I eat *laswa* especially *tugabang* a slippery type green leafy vegetable that my mother and *paltera* (traditional birth attendant) believes will help with easy delivery and my friends who had babies also did.

In Zambia, mothers were encouraged to eat locally defined nourishing foods, such as cooked vegetables with pounded groundnuts and *nshima* (maize flour) [41].

Many foods were proscribed in pregnancy, often those that were classified as ‘cold’. In Pakistan, recent mothers were advised “not to eat rice, prawns and fish” since it would cause lower abdominal pain [16, 51]. On the other hand, women were advised to eat specific types of food in order to bleed heavily since women perceived heavy post-partum bleeding as “healthy since it releases the impurities from the woman’s stomach.” An educated informant from urban Karachi with two children stated [51]:

*Goandh* (sweet made out of semolina, sugar, clarified butter and nuts) is eaten in order to bleed heavily. Also, turmeric powder, dried dates in milk and herbs help one to bleed heavily.

Most fruit and vegetables were thought to be cold; the effects of eating ‘cold’ food include diarrhoea in baby and mother, body swelling, stomach discomfort, aches and pains and cough [48]. South Asian women also believed that ‘cold’ foods, such as yogurt during the puerperium can have long-term negative health consequences including “body aches, weakness and fever” [51]. Foods considered taboo post-partum in rural Bengal were: certain varieties of green leafy vegetables, fibrous vegetables, melons, gourds, pumpkin, papaya, eggplant, shell fish, eggs, lemons, limes, oranges, grapes, chillies, bell peppers, spices, bananas, yoghurt, and oily food [31].

In Nepal, green vegetables, pumpkins and apples were considered to be cold food and believed to cause diarrhoea for the child through mother’s milk, therefore restricted for two to three months after delivery [52]. Khmer women were in fact so concerned of the high risk of *pit duen* (emic term for post-partum sickness) which is felt to be food related, that they end up with a severely restricted diet, as many taboo foods are part of everyday diets [27]. Traditional birth attendants in Zambia advised expectant mothers not to eat eggs as “the baby will be born without hair,” to avoid fish as this can “cause infant abnormality” [41]. There was little consistency in what foods were considered helpful or harmful, even within small rural populations in the same country. For example in rural Laos, some considered that eating coconut or pork would make the baby fat and cause a difficult birth; in an adjacent village, eating coconut was thought to make delivery easier [27].

### Restrictive practices during perinatal period influencing maternal nutrition: “They don’t let you eat, you can’t eat salt, oil, …you can’t eat your fill” [53]

Restrictive practices in the perinatal period were widespread across Asia, Africa and parts of Latin America and involved food, physical activity including sexual intercourse and women’s mobility. Cultural taboos related to food avoidance of ‘bad’ food and restriction went hand in hand and was particularly prevalent and persistent in Asia. As this quote from a mother in rural Bengal (India), restriction of both quantity and type of food was practised [31]:

After delivery for the first three days I was on a diet of dry food, such as rice crisps, garlic, and ghee, and was allowed to eat only once a day, as this diet helps to contract the uterus quickly.

Strict home confinement post-partum common in south-east Asia, was usually spent at the natal home, and could last from seven days to 41 days. The Chinese practice of *zuo yuezi* or ‘doing the month’ (although the period may stretch beyond the month) is intended for the mother to recover her physical strength after birth. While Chinese mothers were aware of the need to enhance their nutrition, during *zuo yuezi* food was restricted to easily digestible foods such as millet soup, brown sugar water and eggs [53]. Due to the severe food restrictions and lack of sunlight as women were confined to a dark room, women can grow weak over the time period, “after 40 days, I couldn’t even stand up!” as one post-partum woman in rural China reported [53].

Women in Myanmar like their counterparts in China abstained from sex, avoided exposure to wind and cold, handling soap, or strenuous house hold chores. They shunned the smell of frying or burning as it could “cause puffiness of the face,” avoided quarrelling, crying, reading and watching television/video [39]. For Khmer women, who have many emic terms for perinatal health/wellbeing states, relapse from food appeared to be most common type of “*toas*” (post-partum state with symptoms including headache, diarrhoea, weakness, palpitations, abdominal pains or cramps, and poor appetite), leading to many prohibitions during the postpartum period. Foods considered particularly dangerous for postpartum women included pineapple, jackfruit, different varieties of bananas, field cucumbers, buffalo meat, pig’s head, and different varieties of fish [40]. Bangladeshi women like their South Asian counterparts reported rigidly controlled diets after birth; they were advised to eat dry food, and rice with mashed potato and black cumin seed; to “cool women’s stomachs and promote production of breast milk” [54].

In parts of Africa, there were strong restrictions on mobility, activity and food during pregnancy; the foods to be avoided often included locally available, protein and energy-rich sources. In Nigeria, the traditional healer was often the first person to ‘diagnose’ the pregnancy, and would proscribe various activities once pregnancy was confirmed including moving about after dark and eating a range of foods such as okra, snails, bush meat, peanuts and bananas [55]. Indigenous women from Brazil also practiced strict confinement or *resguardo* after childbirth with food restrictions; lasting for 40 days. Taboo foods included certain fish, monkeys, tapir, caiman, some turtles, wild pigs and numerous fruits; these make mothers *faz mal* [sick] [56].

### Cross-cutting themes

The following themes seemed to underpin maternal-infant nutritional practices across geographical regions, reflecting society-wide aspects. There were striking similarities across studies from disparate geographical regions and settings.

### Role of woman/mother/wife as strong and good: “I guess as women we just bear that burden” [53]

In many traditional societies, a woman’s worth is pegged to her performance of culturally defined reproductive and productive roles. Similar to other groups for example, the Sabiny (Uganda), regarded motherhood as one of the most important attributes defining a married woman and hard work and endurance were synonymous with pregnancy and childbirth [57]. Coping with severe restrictions and bearing pain throughout the perinatal period were considered to demonstrate strength in mothers. Thus post-partum Khmer women for example would stick to a very restricted diet of salt and galangal up to one month even though some may be crying with hunger [27].

### Poverty and its pervasive effects on perinatal nutrition: “We mostly eat the cheapest available food” [29]

For many childbearing women in low resource settings, knowledge of good nutrition in the perinatal period, whether culturally sanctioned or promoted by healthcare workers could not be put into practice. Ghanaian mothers admitted that although they knew the importance of vegetables, they could not afford to buy them [58], Argentinian mothers similarly knew about good perinatal nutrition, but only “when I can afford them” [59]. Pregnant Kenyan women knew what ‘good’ foods were, but consumed just carbohydrates as they could not afford good food regularly [45]. In rural Bengal, women who had any ‘special’ food, were from upper socioeconomic backgrounds and women who had delivered at their natal homes [31]. Some women simply could not “afford the cost of eating more” during the critical perinatal period [33].

### Change is constant but direction of change unpredictable: “But now we have forgotten our herbs because there are hospitals around” [36]

This theme was evident in the changing breastfeeding practices in spite of strong cultural support. For example, Chinese women acknowledged the nutritional value of breast milk as it promoted “immunity in the baby; makes the uterus smaller; ….. helps the relationship between the mother and baby” [48]. Exclusive breastfeeding was however rare and supplementing common. Even in well supported programs, mothers in South Asia, reported knowing about colostrum and its benefits for babies, “but this knowledge not translated properly into practice” [60].

The studies examined displayed cultural practice being dynamic, however, as this quote from a Ghanaian woman suggests, change in cultural practice may come at a price [36]:

But now we have forgotten our herbs because there are hospitals around. Most of the herbs can't even be found today, and we don't know the laws of the herbs. The hospital is good, but most of us can’t afford to go there.

It was not unusual for pregnant or lactating mothers, supported by their families, to supplement western medicines with traditional medicines. As expressed by this Ghanaian mother, “I made sure that I took all the drugs they gave me at the clinic. … at times, too, I prepared herbs …and added it to the drugs” [61]. The practice of “doing the month” is also morphing slowly, now women may eat “rice porridge, noon a bowl of noodles and two eggs, maybe two slivers of vegetables…. In the past, all the mother could do was drink millet soup” [53]. Contemporary Swazi mothers might resist using traditional medicines during pregnancy because they do not believe in them; in the postpartum period however, they use traditional medicines on their babies as they surrender to the cultural authority of their in-laws [62]. Some Ghanaian women also resist traditional herbs as “I’m afraid [herbs would] abort the baby” [38].

There was evidence of positive change in breastfeeding practice although slow, “I didn’t give the baby anything until the breast milk came. I just kept on putting him on the breast” (older mother, Uganda, one of small minority) [34].

## Discussion

Our review and synthesis of qualitative studies exploring the perinatal period in low resource settings found that cultural influences on maternal and infant nutrition remain strong and they extend throughout the perinatal continuum. The richness and diversity of cultural practices that continue to influence perinatal nutrition both in urban *and* rural settings across LMICs, supports our view that generalisation is not possible and comparison between groups and regions is in fact odious. It is likely that cultural beliefs and understanding exert a stronger influence on maternal behaviour in relation to nutrition, than biomedicine. There is strong cultural support for many health promoting and life-giving nutrition practices such as breastfeeding, and the acknowledgement of the inherent healing properties of foods. Change is occurring in cultural practices and sometimes rapidly, but there is an inherent tension in incorporating ‘positive’ behaviour change versus resisting change. As Kaphle et al. [63], note with respect to childbirth traditions in Nepal, there is a schism between medical and cultural understanding; biomedicine needs to “understand and accommodate culture and tradition.”

While we found strong cultural support and perhaps ambivalent support from elders for breastfeeding, it may no longer be seen as the norm [64]. Optimum breastfeeding is under threat globally, despite robust evidence for exclusive breastfeeding [65] and sound infant and young child feeding strategies being promoted [66]; with only a third of infants worldwide exclusively breastfed for the first four months of life [67]. We know that many upstream determinants such as poverty, livelihood and living arrangements, as well as poor social support and poor knowledge, socio-cultural factors, myths and misconceptions affect breastfeeding duration in low resource settings [68, 69]. With rapid demographic change, practices are changing and not always in the direction expected. For example in the *Tsimane* tribe in Bolivia, contrary to expectations breastfeeding is most intensive in the most modernized villages [70].

The range of foods considered culturally supported and ‘good’ versus ‘bad’ through the perinatal period may appear bewildering, but health professionals need to acknowledge that notions of heating and cooling foods have far more currency across Asia than bio-medically supported nutrition choices [71]. There were certainly contradictions between regions and population groups and often a lack of consistency between populations living in close proximity, about good and bad foods for mother and infant. What is worth celebrating and building on however, is the cultural acknowledgement of the healing role of local foods.

Of greatest concern for maternal and infant wellbeing, were the widespread and still current, restrictive practices curtailing women’s mobility and nutrition. In fact more foods were restricted and considered taboo, than were promoted or allowed. While the origin of the post-partum confinement practices such as *resguardo* and *zuo yuezi* have their bases in restoring women’s health and strength, they might conversely do the opposite. The cultural underpinning of women’s role as wife, mother and reproducer and therefore needing to ‘bear’ suffering and hardship appear so strong as to negate the life affirming and health promoting aspects of other practices in this period. Studies in South Asia and Africa have shown that with greater access to biomedical health services and modernisation, gender-power inequalities may even increase, with women being subjected to more restrictions and control than men are [72, 73].

We found a paucity of studies from Latin America and the middle-east in this review. This may be due to restricting our literature review to studies published in English. There are far more studies being published in the current decade and the majority of studies emanate from the regions with the greatest burden in maternal and child morbidity/mortality. Nevertheless it would be worthwhile to explore whether the themes we identified have similar currency or transferability across other regions within LMIC. Our perception from the review is that there is a tendency for studies to report mainly negative (or bio-medically ‘frowned upon’) traditional practices.

## Conclusions

We believe that more attention needs to be paid to the important dimension of culture in influencing perinatal nutrition. There is an urgent need for more inter-disciplinary research into socio-cultural factors influencing perinatal nutrition that is readily available for practitioners and policymakers. Whilst attempts to unpack the reasons behind the alarming rates of maternal and child malnutrition in South Asia and sub Saharan Africa are underway, attention needs to be paid to the role of culture in influencing not only women’s choices in nutrition, but other inter-related health behaviour throughout the perinatal continuum. The gendered expectation of women’s roles as reproducers and the influence of restrictions on women’s mobility and access to appropriate nutrition need to be acknowledged in the context of upstream factors such as poverty. Contextually specific, culturally responsive perinatal nutrition interventions therefore are needed to optimise health and wellbeing of mother-infant dyads. Maternal and infant nutrition messages need to incorporate local understanding, such as that of ‘hot’ and ‘cold’ foods, while at the same time ensuring the promotion of nutritious, locally available foods. Public health policymakers and clinicians need to design interventions that build on culturally sanctioned life affirming behaviour such as breastfeeding, promoting post-partum rest and recovery, while modifying the harmful aspects of other perinatal cultural practices; always acknowledging the centrality of culture and understanding that ‘culture is change’.

## Abbreviations

CASP, Critical Appraisal Skills Programme; COREQ, consolidated criteria for reporting qualitative research; LMIC, low and middle income countries; MDG, millennium development goal; PRISMA, preferred reporting items for systematic reviews and meta-analyses

## Additional file

Additional file 1: PRISMA 2009 Checklist. (DOC 63 kb)
